# Chronic hepatitis D infection is associated with distinguishing microbial and functional features in the gut microbiome

**DOI:** 10.3389/fmicb.2026.1851892

**Published:** 2026-07-09

**Authors:** Meital A. Gewirtz, Yue Zhang, Nivedha Vaidy, Neelam R. Redekar, Nicole Minerva, James A. Haddad, Jenna L. Oringher, Rownock Afruza, Moumita Chakraborty, Matthew G. Menkart, Harish Gopalakrishna, Julian Hercun, Justin Lack, David E. Kleiner, Michail S. Lionakis, Christopher Koh, Theo Heller

**Affiliations:** 1 Translational Hepatology Section, Liver Diseases Branch, National Institute of Diabetes and Digestive and Kidney Disease, National Institutes of Health, Bethesda, MD, United States; 2 Integrated Data Sciences Section (IDSS), National Institute of Allergy and Infectious Diseases, National Institutes of Health, Rockville, MD, United States; 3 Laboratory of Pathology, National Cancer Institute, National Institutes of Health, Bethesda, MD, United States; 4 Fungal Pathogenesis Section, Laboratory of Clinical Immunology and Microbiology, National Institute of Allergy and Infectious Diseases, National Institutes of Health, Bethesda, MD, United States

**Keywords:** amino acid, biosynthesis, carbohydrate, energy metabolism, gut–liver axis, metagenome, metatranscriptome, portal vein

## Abstract

**Background:**

The microbiome of patients with hepatitis D virus (HDV) has yet to be characterized. This study aims to (1) characterize gut microbial composition in HDV, (2) determine its functional profile, (3) identify microbial species that contribute to changes in pathway expression, and (4) correlate the changes in the gut microbiome with clinical markers of disease severity.

**Methods:**

Cross-sectional analyses of 35 HDV-infected patients and 32 healthy controls (HCs) were performed. DNA and RNA were isolated from stool and sequenced by shotgun-sequencing. Microbial and functional profiles were compared between the HDV-cohort and HCs to identify disease-specific alterations to the gut microbiome. Clinical metadata were used to identify correlations with disease severity.

**Results:**

There were significant changes in the composition of the gut microbiome in HDV-infected patients as compared with HCs, spanning multiple bacterial phyla. Expression of 194 pathways was significantly increased in the HDV group. Pathways that were upregulated in the HDV cohort were related to amino acid and carbohydrate biosynthesis or involved important metabolic cofactors and carriers. Several microbial species, including *Bacteroides fragilis*, *Cateibacterium mitsuokai*, and *Faecalibacterium prausnitzii*, were identified as contributing to the differentially expressed pathways. Four genera correlated with hepatic venous pressure gradient (HVPG).

**Conclusion:**

There are significant differences in microbial composition between HDV and HCs, several of which are found to be altered in other liver diseases. Upregulated pathways suggest a broader dysregulation of energy metabolism, even in early disease. These findings provide insight into pathways that may lead to liver disease progression in HDV.

## Introduction

Hepatitis D virus (HDV) coinfects with the hepatitis B virus (HBV) and causes the most severe form of chronic viral hepatitis. HDV infection occurs in an estimated 15–20 million people globally and is a common cause of cirrhosis and hepatocellular carcinoma (HCC) in HBV-infected individuals (Chen et al., 2019).

The gut microbiome, a dynamic community of microorganisms, plays an important role in host metabolic and immune functions (Padilha et al., 2025). The commensal bacteria residing in the human gut can encode a vast array of genes, many of which perform functions that the host lacks the capability to do on its own. Although gut bacteria and their human host have evolved a symbiotic relationship, certain bacterial species are associated with disease. Given the bidirectional communication between the gut and the liver through the portal vein, biliary tract, and systemic circulation, the gut microbiome, specifically gut dysbiosis, has been hypothesized to contribute to the pathogenesis of various liver diseases (Wang et al., 2021; Jiang et al., 2015; Caussy et al., 2019; Dubinkina et al., 2017; Leclercq et al., 2014; Shen et al., 2023). Many of these studies infer the functional potential from the changes in microbial composition. The gut microbiome has been studied in the context of a wide range of liver diseases, including metabolic dysfunction-associated steatotic liver disease (MASLD) (Jiang et al., 2015; Caussy et al., 2019; Nobili et al., 2018), alcohol-associated liver disease (Dubinkina et al., 2017; Leclercq et al., 2014), and viral hepatitides (Leclercq et al., 2014; Aly et al., 2016; Shen et al., 2023; Li et al., 2022).

To our knowledge, the gut microbiome in HDV patients has not yet been thoroughly characterized. In this study, the composition and functional profile of the gut microbiome were characterized through metagenome and metatranscriptome shotgun sequencing. We identified significant compositional differences across several bacterial phyla and various functional differences in the gut microbiome of HDV patients, which correlated with markers of liver disease severity.

## Materials and methods

### Study population

Thirty-five adult subjects (age > 18 years) with chronic HDV infection, confirmed by the presence of quantifiable HDV RNA (>2log10 above the lower limit of quantification) in serum, were recruited for study #NCT02511431 and #NCT03600714. Chronicity was established by either presence of HDV RNA in serum for more than 6 months or the presence of anti-HDV antibodies for more than 6 months. Prior to enrollment all subjects underwent a comprehensive evaluation to exclude other chronic liver diseases. In addition, exclusion criteria included decompensated liver disease (defined by bilirubin >4 mg/dL, albumin <3.0 gm/dL, prothrombin time >2 s prolonged, or a history of bleeding esophageal varices, ascites, or hepatic encephalopathy), pregnancy, breastfeeding, immunosuppressive therapy, recent experimental or pegylated interferon treatment (within 6 months), active alcohol consumption, autoimmune conditions, malignancy, and HIV infection. All subjects received HBV treatment with nucleos(t)ide analogues. At the baseline visit, subjects underwent transjugular liver biopsy with portal pressure measurements. Liver biopsies were scored for hepatic necroinflammation using the histologic activity index (HAI) scoring system and for fibrosis using the Ishak Fibrosis (IF) scoring system. All biopsies were scored by a single expert pathologist. Thirty-two healthy controls (HCs) were recruited for studies #NCT01386437 and #NCT01915251 in conjunction with The Fungal Pathogenesis Section of NIAID.

### Sample collection

**Figure 1:**
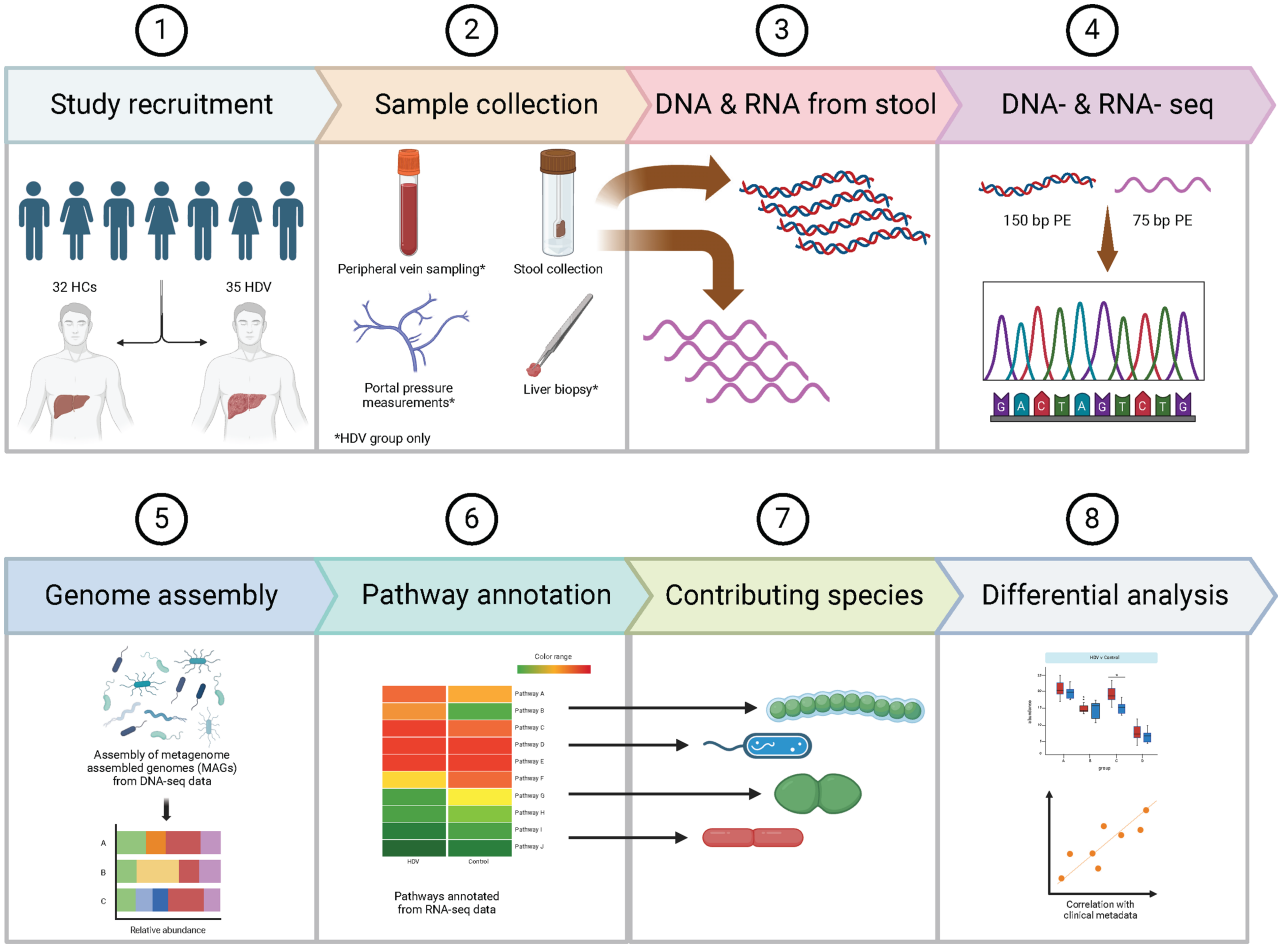
Study design. 32 HCs and 35 HDV patients were recruited to the study (1). Stool was collected from all participants. In order to examine correlations with clinical metadata within the HDV group, liver biopsy, portal pressure measurements, and peripheral blood was also collected from HDV patients (2). DNA and RNA were extracted (3) and subjected to shotgun sequencing (4). Genomes were assembled from DNA-seq data (5) and transcriptomes were assembled from RNA-seq data (6). Species responsible for transcriptomic differences were identified by mapping the RNA-seq data back to the DNA-seq data (7). Differences between the HDV and HC groups and within the HDV group based on clinical metadata were identified using genomic and transcriptomic analyses (8). Created in BioRender. Gewirtz (2024) https://BioRender.com/o85t899.

Study design is described in Figure 1. Only samples collected during the baseline visit prior to initiation of trial medication were utilized for this analysis. Prior to stool sample collection, subjects adhered to their regular diets, and their dietary intake on the day preceding stool collection was documented. Stool samples were processed within 4 h of collection, where they were portioned into 250–500 mg aliquots without mixing, with each aliquot distributed into three cryovials and preserved at −80 °C until nucleic acid extraction. The samples were not preserved in DNA or RNA shields.

### DNA isolation and whole metagenome shotgun sequencing

DNA was isolated using the Qiaamp powerfecal pro DNA kit (Qiagen #51804). Following extraction, DNA was purified using AMPure XP beads. DNA libraries were prepared using Nextera DNA Flex Library Prep (Illumina #20018705) and IDT for Illumina Nextera DNA Unique Dual Indexes Set A (Illumina # 202027213). Prior to sequencing, libraries were quantified using the PicoGreen dsDNA assay and quality was assessed using the Bioanalyzer 2100 High Sensitivity DNA assay. Sequencing was done over two lanes of the Illumina NovaSeq 6000 (150bp x2).

### RNA isolation and whole metatranscriptome shotgun sequencing

RNA was isolated using a modified phenol-chloroform extraction. Briefly, frozen stool was combined with silica beads, TE buffer, SDS, and acid phenol and vortexed. Samples were homogenized using the TissueLyzer II at 25 Hz for 40s and then cooled on ice for 90s (3x). Samples were centrifuged at 13,400 *g* at 4 °C for 15 min. The aqueous phase was combined with 5:1 phenol:chloroform isoamylalcohol and vortexed for 2 min. Samples were centrifuged at 13,400 *g* at 4 °C for 5 min and the aqueous phase was isolated. Addition of the phenol-chloroform mixture and centrifugation steps were repeated 3 times. Next, the aqueous phase was combined with sodium acetate, glycogen, and ethanol. Samples were frozen at −80 °C for at least 1 h. Samples were centrifuged at 13,400 *g* at 4C for 15 min and the supernatant was discarded. The pellet was washed with 70% EtOH, dried for 15 min at RT and then resuspended in RNAse free water. Next, Ambion Turbo DNA-free kit was used (Invitrogen catalog no. Am1907) to remove DNA following manufacturer’s instructions. RNA was further purified using the Qiagen RNeasy kit (catalog no. 74104) following manufacturer’s instructions. The concentrations and quality (RIN) were assessed using the BioAnalzyer 2,100. rRNA was depleted and libraries were prepared with NEBNext rRNA Depletion Kit (Bacteria) (NEB #E7860) and NEBNext Ultra II Directional RNA Library Prep Kit for Illumina (NEB #E7765) using NEBNext Multiplex Oligos for Illumina 96 Index Primers (E6609S). Libraries were sequenced over two lanes of the NovaSeq 6000 (75bp x2).

### Metagenomic and metatranscriptomic read preprocessing

Raw DNA reads were trimmed for low-quality and adaptor regions and decontaminated for human reads using the read_qc module (trim_galore v0.5.0 and BMTagger v3.101) in the metaWRAP pipeline (v1.2.1) (Uritskiy et al., 2024). Raw RNA reads were quality-checked with FastQC (Brown et al., 2017) (v0.11.8) and decontaminated to remove host (human) reads using BMTagger (v3.101). A total of 33.1 ± 7.7 million DNA and 70.5 ± 16.1 million RNA paired-end reads were retained. Raw sequencing data are deposited in NCBI SRA (accession number: PRJNA1144402).

### Generation of metagenome-assembled genomes

The preprocessed DNA reads were assembled into contigs in a sample-independent manner using metaWRAP assembly module with metaSPAdes (Nurk et al., 2017) (v3.13.0) and megaHIT (Li et al., 2016) (v1.1.3) assemblers. Within each sample, the contigs were binned to generate draft bin sets with metaWRAP binning module. The binning module calls MetaBAT2 (Kang et al., 2015) (v2.12.1), MaxBin2 (Wu et al., 2026) (v2.2.6), and CONCOCT (Alneberg et al., 2014) (v1.0.0) to generate three sets of draft bins. The bin sets were then consolidated into one bin set with completeness >50% and contamination level <5% using the metaWRAP bin_refinement module. We identified 2,034 and 1,618 refined genomic bins from HDV and control groups, respectively, with similar bin characteristics (Supplementary Figure 1). The refined bin sets of all samples were merged, and redundant genomic bins were removed using the dereplicate function in dRep (Olm et al., 2017) (v3.2.2) with options “-l 10,000 -pa 0.9 -sa 0.95 -nc 0.8 -cm larger” to extract a non-redundant bin set.

### Taxonomic and functional annotation

The metagenomic reads were mapped to metagenome-assembled genomes (MAGs) using bbmap (bbtools suite v38.87) and annotated for taxonomy and functions. They were imported into MetaPhlAn3 (Beghini et al., 2021) (v3.0) for taxonomic classification at the species resolution. The normalized count of classified reads was collapsed at every taxonomic level. Functional profiling was obtained using HUMAnN3 (Beghini et al., 2021) (v3.0), which classifies and groups reads into MetaCyc pathways (Caspi et al., 2016) and calculates their abundance at the community and species-level (normalized to copies per million). To annotate the metatranscriptomic data, HUMAnN3 (Beghini et al., 2021) was run with the taxonomic profile of the corresponding metagenomic data to ensure that RNA reads were mapped to any pangenomes detected in the metagenome (using the –taxonomic-profile option). The RNA pathway abundance count matrix is normalized to copies per million before downstream.

analysis.

### Microbial community diversity analysis

Alpha [richness and Shannon’s (Shannon and Weaver, 1949)] and beta diversity metrics [weighted and unweighted UniFrac distance (Chen et al., 2012)] were calculated using MetaPhlAn3’s (Beghini et al., 2021) auxiliary script calculate_diversity.R with the relative abundance table at the species level. Significance in alpha diversity was tested with the Wilcoxon rank sum test. To compare the microbial community composition at the group level, permutational multivariate analysis of variance [PERMANOVA (Anderson, 2001)] was used to probe the difference in the centroid locations between HDV and control in the UniFrac distance metrics. Permutational multivariate analysis of group dispersion homogeneity [PERMDISP (Anderson et al., 2006)] was applied to compare the within-group dispersion level between groups in the UniFrac distance metrics.

### Differential abundance analysis

The metagenomic abundance of taxa and pathways, and the metatranscriptomic abundance of pathways were subjected to differential abundance analysis. Briefly, MaAsLin2 (Mallick et al., 2021) (v1.12.0) was used to screen taxa and pathways significantly associated with HDV status or correlated with clinical metadata (described below). Using MaAsLin2, a general linear model was applied to correlate the log-transformed relative abundance of each taxon or pathway with a predictor of interest (as described below) while adjusting for age, BMI and sex as covariates. Only features present in >10% of the number of samples of each test were selected for testing.

Taxa and community-level pathways were first tested for their associations with HDV status. Taxa and pathways significantly associated with HDV (adjusted *p* value <0.1, Benjamini–Hochberg procedure) were regressed individually against Ishak Fibrosis score, HAI, portal pressure, platelet counts, disease severity (mild or severe), and inflammation severity (mild or severe). Furthermore, community-level pathways significantly associated with HDV (adjusted *p*-value <0.1) were stratified to the species-level to find species-level pathways associated with HDV. Similarly, any species-level pathways significantly associated with HDV (adjusted *p* value <0.1) were correlated with the clinical metadata.

### Differential expression analysis

Differential expression analysis reveals how regulation or expression of pathways is associated with disease. We applied MTX_model (Zhang et al., 2021) (v1.2.2) to test associations between pathway expression and HDV status as well as clinical metadata. MTX_model adjusts for metagenome abundance by including the metagenome relative abundance of a given feature as a continuous covariate in the differential expression model. We applied the same procedure described in differential abundance testing to find the differentially expressed community- and species-level pathways associated with HDV or clinical metadata.

### Statistical analysis and data visualization

All downstream analyses were performed in R (v4.2). PERMANOVA and PERMDISP were performed with R package metagMisc (v0.0.4). Wilcoxon rank sum test was performed with rstatix (v0.7.2). clPlots were generated with ggpubr (v0.6.0), ggplot2 (v3.4.2), and ComplexHeatmap (v2.14.0). Data analysis scripts are publicly available on GitHub at https://github.com/GaryZhangYue/Gewirtz_et_al_HDV_Microbiome.

## Results

### Study cohort

The study cohort consisted of patients with chronic HDV infection (*n* = 35) and healthy controls (*n* = 32) who were included for microbiome analysis. Baseline demographic and clinical characteristics of the cohort are summarized in Table 1.

**Table 1:** Characteristics of study population.

Variables	Control	HDV	*p*-value
Total number of DNA samples	32	35	
Total number of RNA samples	32	29	
Age (years)	38.7 ± 12.9	41.5 ± 9.6	0.32
Sex (M)	15	20	0.55
Race (*n*)			
White	22	11	
Black/African American	2	5	
Asian	5	18	
Multiracial	2	1	
Other	1	0	
Country of origin (*n*)			
United States of America	19	3	
Pakistan	1		
Guatemala	1		
Bolivia	1		
Venezuela	1		
Egypt	1		
China	2	1	
Columbia	1		
Greece	2		
Portugal	1		
Canada	1		
Malta	1		
Cameroon		1	
Mongolia		15	
Sierra Leona		1	
Russia		5	
Moldova		2	
Ukraine		1	
Romania		2	
Togo		1	
Liberia		1	
Ivory Coast		1	
Myanmar		1	
HDV genotype (*n*)			
1		30	
5		3	
Not documented		2	
Body Mass Index (BMI) (Kg/M2)	24.1 ± 4.4	25.7 ± 4.1	0.13
Alkaline phosphatase (U/L)		77 (40–188)	
ALT (U/L)		88 (26–348)	
AST (U/L)		57 (20–197)	
Total bilirubin (mg/dL)		0.4 (0.2–1)	
Albumin (g/dL)		3.94 (3.2–4.8)	
Platelets (10^9/L)		174 ± 56	
Ishak fibrosis score		3.1 ± 1.5	
Hepatic activity index total (0–18)		8.7 ± 2.1	
HDV RNA (log10 genome equivalents/mL)		5.36 (2.91–9.13)	
Hepatic venous pressure gradient (mm Hg)		4.7 ± 2.1	

Age, sex, and BMI were not significantly different between the two groups. *p*-values for age and BMI were calculated using a *t*-test and *p*-value for sex was calculated using a chi-squared test. HDV patients were predominantly noncirrhotic with an average Ishak fibrosis score of 3.1.

### Major taxonomic groups in study cohort

**Figure 2:**
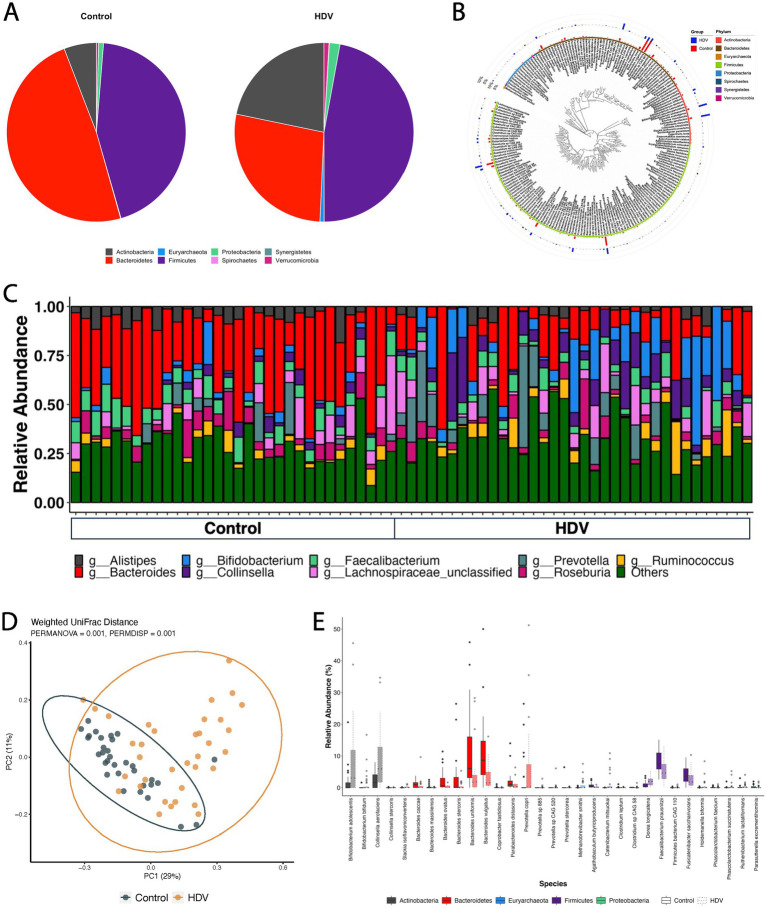
The microbial composition of the gut microbiome was different in the HDV group compared with the control. **(A)** Pie charts illustrating the top 8 phyla within the gut microbiomes of each cohort. Actinobacteria, Bacteroidetes, and Euryarchaeota were significantly different between the two groups. **(B)** Phylogenetic tree of the species represented in the gut microbiome of HDV and control groups. Relative abundance of each species is depicted in red (control) or blue (HDV). **(C)** The HDV group had higher variability in microbial composition compared with HCs. **(D)** PCoA of Weighted UniFrac Distance have distinct clusters with different centroid locations (Permanova = 0.001, PERMDISP = 0.001), demonstrating that the relative abundance differs significantly between groups. **(E)** Box plot depicting the 31 species that were significantly different between the two groups after FDR corrections (*q* < 0.1). Species are grouped by phyla and stars indicate the level of significance (^*^q < 0.1, ^**^q < 0.05, ^***^q < 0.01).

We identified a total of 983 non-redundant MAGs. Collapsing the taxa at the phylum level, we observed a total of eight phyla, in which *Bacteriodetes*, *Firmicutes* and *Actinobacteria* were dominant in relative abundance across all samples (Figure 2A; Supplementary Figure 2). We classified a total of 206 species in HDV and HC combined (Figure 2B). *Firmicutes* had the highest number of species classified, followed by *Bacteroides* and *Actinobacteria*. Dominant species included *Prevotella copri*, *Bacteroides vulgatus*, *Bacteroides uniformis*, *Bifidobacterium adolescentis*, *Collinsella aerofaciens*, *Faecalibacterium prausnitzii*, and *Eubacterium rectale*. In comparison with HC, HDV group had higher variability in microbial composition (Figure 2C).

### Significant differences in gut microbiota between HDV-patients and HCs

To explore the differences in microbial diversity between HDV and HCs, we analyzed the alpha and beta diversity metrics based on the relative abundance of the classified species. There were no significant differences in richness (i.e., the number of identified species) or Shannon index observed between groups (Supplementary Figure 3) at significance threshold of 0.05. Significant between-group differences in beta diversity in centroid locations (PERMANOVA, *p* = 0.001) were observed, however, in both unweighted and weighted UniFrac distance metrics, even if the two groups overlapped in the PCoA plots (Figure 2D). This suggests that within the identified species, the microbial community composition was significantly different on the group-level, although several samples in the two groups shared a similar microbial community structure since they were located adjacent to each other on the PCoA plot. Furthermore, HDV had a significantly higher level of within-group variability compared to HCs in the weighted UniFrac metrics (PERMDISP, *p* = 0.001). The diversity analysis suggested a significantly more variable microbial community structure in the HDV group compared to HCs.

### Taxonomic groups associated with HDV and clinical metadata

To identify associations between disease and gut microbiota composition, we tested differentially abundant taxa. Among 206 identified species, 31 species were significantly associated with HDV status (*q* < 0.1, Figure 2E). Among those, *Bacteroides uniformis*, *Bacteroides vulgatus*, *Faecalibacterium prausnitzii*, and *Fusicatenibacter saccharivorans* were dominant in HC and had a significantly lower relative abundance in HDV. On the other hand, *Bifidobacterium adolescentis*, *Collinsella aerofaciens*, and *Prevotella copri*, while relatively scarce in controls, had a significantly higher abundance in HDV.

To investigate how species within the same genus combined to vary in association with HDV and HC, we collapsed the relative abundance at the genus level and reiterated the association analysis as above. The genera *Catenibacterium*, *Slackia*, *Dorea*, *Collinsella*, *Prevotella*, *Holdemanella*, *Coprococcus*, *Methanobrevibacter*, *Lactobacillus*, and *Ruthenibacterium* were significantly increased in the HDV group compared to HCs (*q* < 0.1). The genera *Bacteroides*, *Parabacteroides*, *Faecalibacterium*, *Parasutterella*, *Coprobacter*, *Flavonifractor*, *Alistipes*, *Agathobaculum*, *Barnesiella*, and *Fusicatenibacter* were significantly decreased in the HDV cohort compared to HCs (*q* < 0.1).

The differences in phylogeny between the groups spanned different phyla (Figure 2A). While the phyla *Actinobacteria* (kingdom Bacteria) and *Euryarchaeota* (kingdom Archaea) were significantly increased in the HDV cohort, the phylum *Bacteroidetes* (kingdom Bacteria) was significantly decreased, as compared to HCs (*q* < 0.1).

Testing for significant differences at each taxonomic level revealed that several families were enriched in the HDV group including, *Bifidobacteriaceae*, *Coriobacteriaceae*, *Enterobacteriaceae*, *Erysipelotrichaceae*, *Lactobacillaceae*, *Methanobacteriaceae*, and *Prevotellaceae*. Five families were reduced in the HDV group. These were *Bacteroidaceae*, *Barnesiellaceae*, *Rikenellaceae*, *Sutterellaceae*, and *Tannerellaceae*.

**Figure 3:**
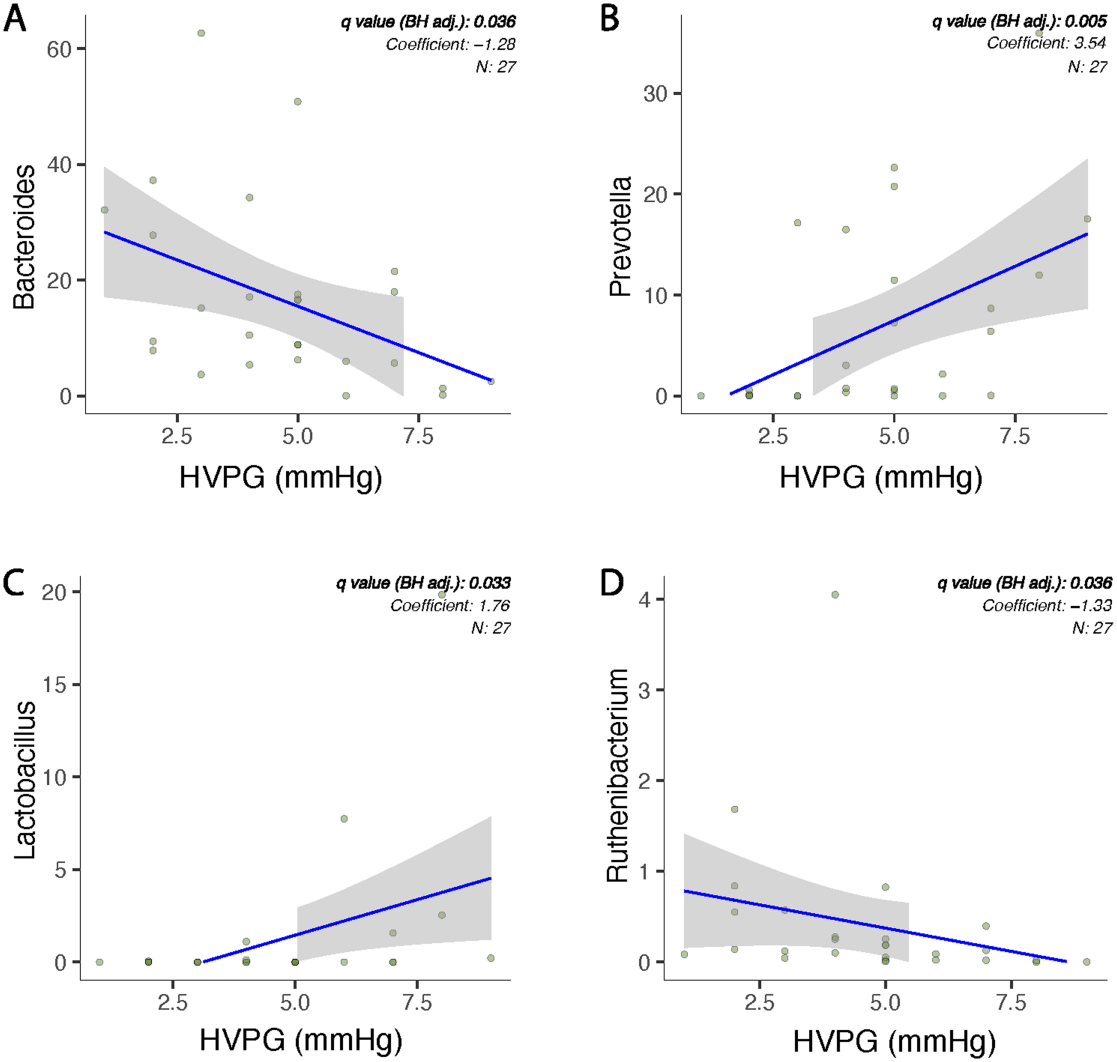
Four genera significantly correlated with portal pressure within the HDV group. These genera were **(A)** *Bacteroides*, **(B)** *Prevotella*, **(C)** *Lactobacillus*, and **(D)** *Ruthenibacterium*. Consistent with previous literature on the subject, Prevotella and Bacteroides have an inverse relatationship. Y-axis shows the relative abundance of genera. The blue line shows the linear regression trend between portal pressure and the relative abundance of the given genus in the normal space. The *q* value (*p* values adjusted by Benjamini–Hochberg procedure), Maaslin2 coefficient (equivalent to log2 fold change), and the number of samples included in the model are indicated on each plot.

To determine if any key clinical parameters were associated with changes in the gut microbiome in HDV patients, we next correlated the relative abundance of the differentially abundant taxa with the clinical metadata. Only HVPG was significantly associated with any changes to the composition of the gut microbiome. The genera *Prevotella* and *Lactobacillus* were positively associated with HVPG. The genera *Bacteroides* and *Ruthenibacterium* were negatively associated with HVPG (Figure 3).

### HDV group had significantly upregulated classified pathways compared to HCs

**Figure 4:**
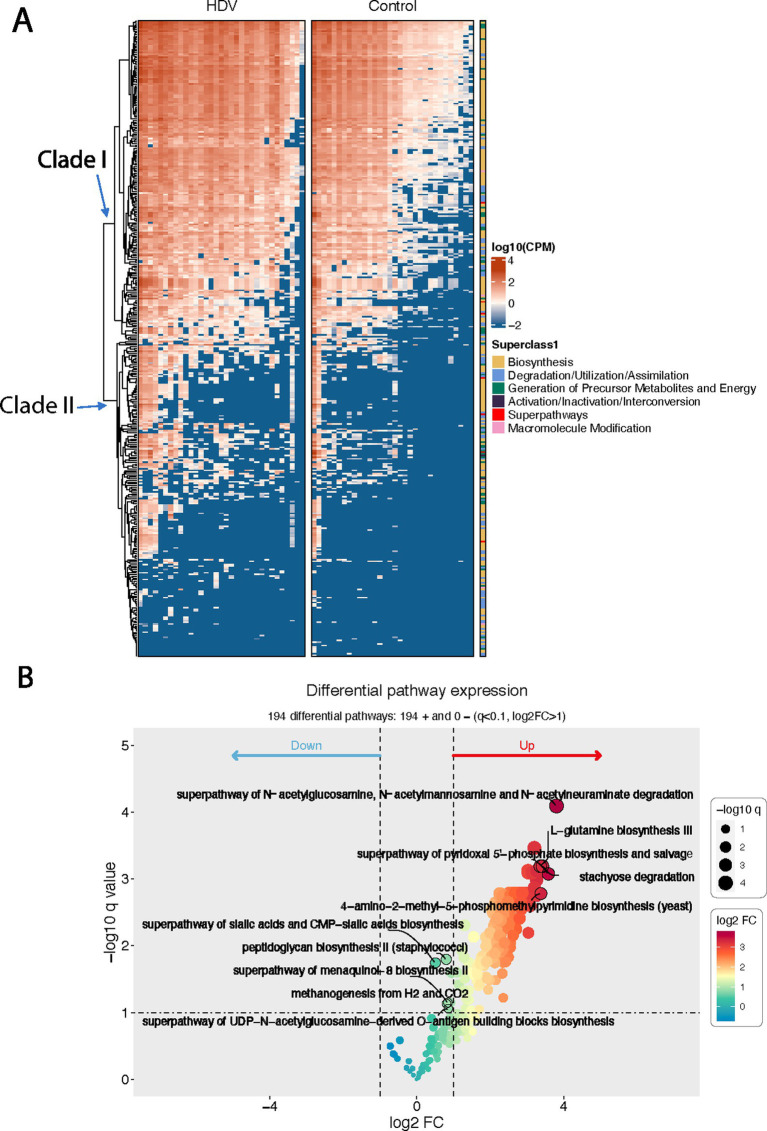
HDV group had significantly upregulated classified pathways. **(A)** 385 community-level pathways were identified. These pathways were divided into six different Superclass1 groups. **(B)** RNA transcript abundance was corrected for DNA abundance using the MTX model, allowing us to determine the expression of significantly different pathways. 194 pathways were significantly upregulated in the HDV group and none were downregulated (*q* < 0.1, log2FC > 1).

To further probe the associations between HDV and gut microbiota functional profiles, we explored the transcriptome variations between HDV and HCs. A total of 385 community-level MetaCyc pathways were identified in metatranscriptome data (Figure 4A, Supplementary Figure 4). On average, the UniRef90 gene classification rates were 81 and 79% for metagenome and metatranscriptome, explaining the majority of the data. The MetaCyc pathway integration rates were 5.7% (metagenome) and 0.73% (metatranscriptome), comparable to a relevant study (Abu-Ali et al., 2018). Biosynthesis, degradation/utilization/assimilation and generation of precursor of metabolites and energy were the major categories of pathways. The heatmap shows a clear segregation pattern of pathways, where first clade was more abundant and prevalent. In particular, the first clade of pathways was highly abundant in most HDV patients, but only abundant in roughly half of the controls.

To investigate the pathways significantly expressed with HDV, we performed general linear model-based differential expression analysis that adjusts for genomic abundance of given pathways using the paired metagenomic data. At the community level, a total of 194 pathways were found to be differentially expressed between conditions, with all of them significantly upregulated in HDV (*q* < 0.1; log2FC > 1; Figure 4B). The predominantly higher expression of identified pathways in HDV is due to their overall higher pathway integration rate in RNA data (1 and 0.5% for HDV and HC on average).

While 42 degradation-related pathways were upregulated, the majority of upregulated pathways were related to biosynthesis (121 pathways), including amino acid biosynthesis (29 pathways), cofactor, carrier, and vitamin biosynthesis (25 pathways), and nucleos(t)ide biosynthesis (23 pathways). The ten most differentially expressed pathways identified were superpathway of N-acetylglucosamine, N-acetylmannosamine and N-acetylneuraminate degradation; purine ribonucleosides degradation; stachyose degradation; L-glutamine biosynthesis III; D-galactose degradation V (Leloir pathway); galactose degradation I (Leloir pathway); L-arginine biosynthesis IV (archaebacteria); L-arginine biosynthesis II (acetyl cycle); L-ornithine biosynthesis; L-arginine biosynthesis I (via L-ornithine) (*q* < 0.0009, logFC>3).

### Species contributing to the differential expression of pathways and their correlation to Ishak fibrosis score

**Figure 5:**
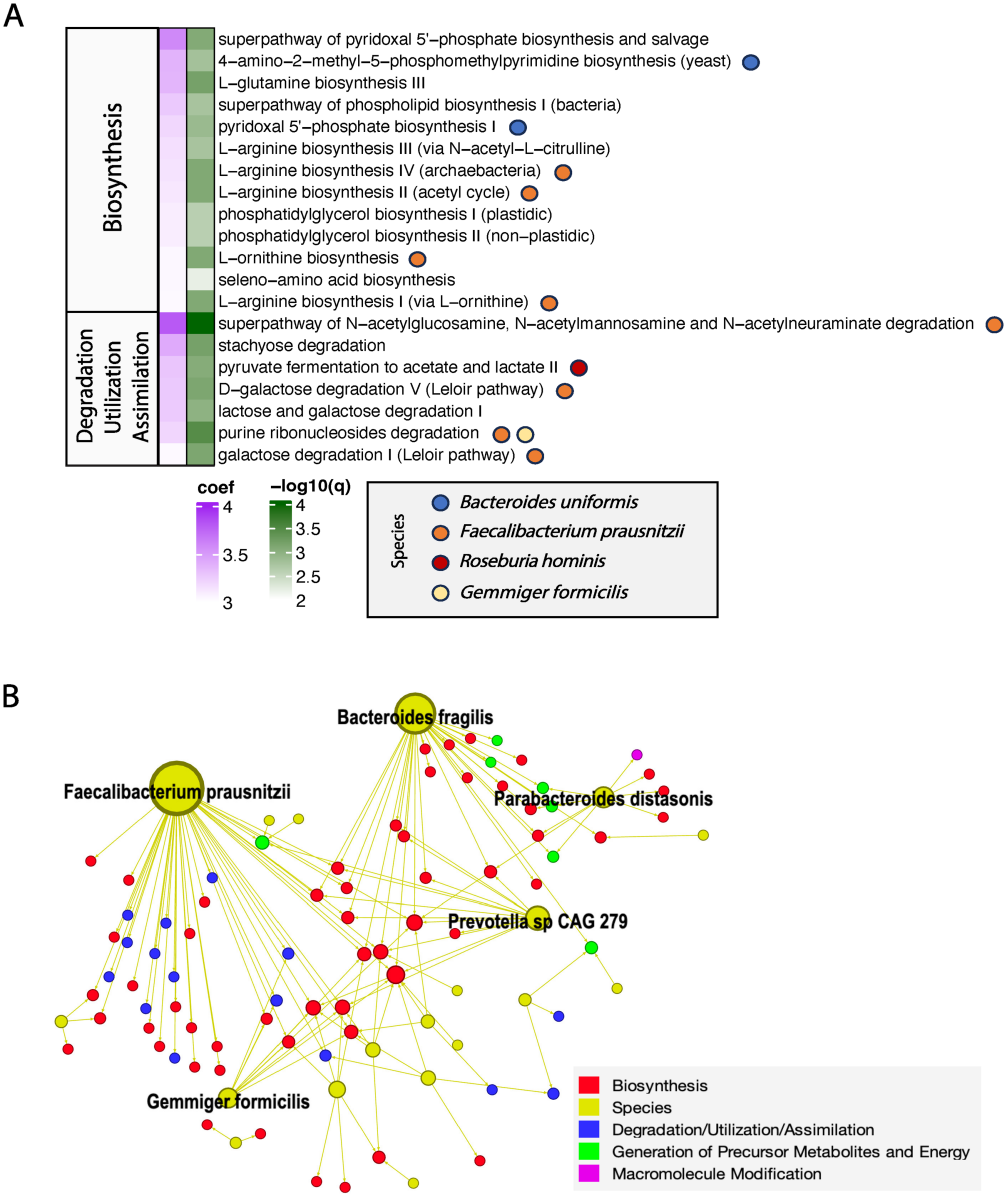
Several species were identified as contributing to the pathways that were significantly different between the two groups. **(A)** Four species were identified as contributing to some of the top 20 significantly differentially expressed pathways. **(B)** Network demonstrating the relationships between bacterial species and the pathways they contributed to. Some species contributed to many pathways.

We next evaluated how differentially expressed pathways at the species-level correlated with HDV metadata. Of the 20 most significantly differentially expressed pathways, four microbial species were identified as contributing to the differential expression of pathways (Figure 5A). *Bacteroides uniformis* contributed to two biosynthesis pathways: 4-amino-2-methyl-5-phosphomethylpyrimidine biosynthesis and pyridoxal 5′-phosphate biosynthesis I. *Faecalibacterium prausnitzii* contributed to 4 biosynthesis pathways and 3 degradation, utilization, and assimilation pathways. *Roseburia hominis* contributed to pyruvate fermentation to acetate and lactate II and *Gemmiger formicilis* contributed to purine ribonucleosides degradation, both degradation, utilization, and assimilation pathways.

Several species were identified as contributing to various pathways in addition to the top 20 pathways; therefore, a network was constructed to better identify the relationships between the various species and pathways (Figure 5B). Microbiota mainly contributed to biosynthesis and degradation, utilization, and assimilation pathways. The species *Faecalibacterium prausnitzii*, *Bacterioides fragilis, Parabacteroides distasonis, Gemmiger formicilis,* and *Prevotella* sp. *CAG 279* were identified as contributing to the largest percentage of differentially expressed pathways.

**Figure 6:**
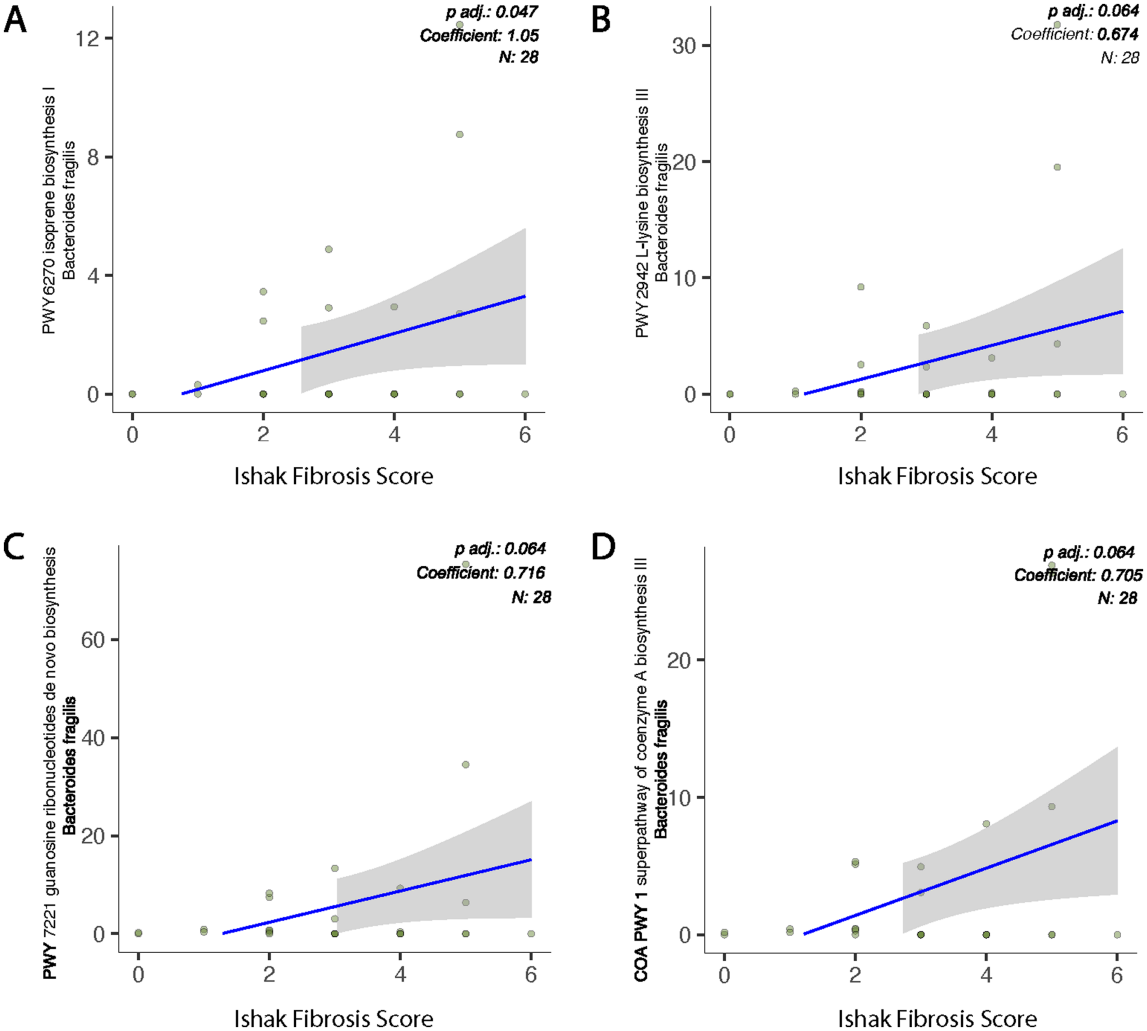
Ishak fibrosis score correlated with the species-level pathway expression of **(A)** Isoprene biosynthesis I (*Bacteroides fragilis*), **(B)** L-lysine biosynthesis III (*Bacteroides fragilis*), **(C)** Guanosine ribonucleotides *de novo* biosynthesis (*Bacteroides fragilis*), and **(D)** Superpathway of coenzyme A biosynthesis III (*Bacteroides fragilis*). Y-axis shows the cpm value of pathways. The blue line shows the linear regression trend between Ishak fibrosis score and pathway cpm value in the normal space. The *p* values adjusted by Benjamini–Hochberg procedure, Maaslin2 coefficient (equivalent to log2 fold change), and the number of samples included in the model are indicated.

Differentially expressed pathways were also correlated to Ishak Fibrosis score. Four pathways at the community level were positively correlated with IF score. These were Isoprene biosynthesis I, guanosine ribonucleotides *de novo* biosynthesis, coenzyme A biosynthesis II, and L-Lysine biosynthesis III (*q* < 0.1), all of which were identified as expression by *B. fragilis* (Figures 6A–D).

## Discussion

The gut–liver axis, which involves the portal vein and the common bile duct, has long been established as integral to the pathogenesis and progression of liver disease (Wang et al., 2021). Microbial products including toxins, metabolites, and short-chain fatty acids (SCFAs) travel from the gut to the liver via the portal vein, while bile acids and other compounds circulate from the liver to the gut via the common bile duct. Accordingly, many studies (Jiang et al., 2015; Caussy et al., 2019; Dubinkina et al., 2017; Leclercq et al., 2014; Shen et al., 2023; Bajaj et al., 2014; Shao et al., 2018) have examined microbial alterations across various liver diseases.

Despite poor outcomes in HDV, including cirrhosis and HCC, the composition and functional profile of the gut microbiome in HDV patients have previously not been characterized. RNA-seq allows for direct evaluation of the microbial transcriptome, a more accurate representation of microbial function than inference from the species identified by metagenomic data alone.

We found that the microbial composition was significantly different between the HDV cohort and HCs, and interestingly, that many of the phyla, genera, and species-level changes have been implicated in liver diseases of other etiologies, as well as in HIV. For example, *Enterobacteriaceae*, which was enriched in the current HDV cohort, was also enriched in alcohol use disorder and alcohol-associated liver disease (Dubinkina et al., 2017) and in NAFLD and NASH (Caussy et al., 2019). Multiple genera such as *Alistipes, Barnesiella, Faecalibacterium,* that were reduced in the current HDV cohort, have also been shown to have lower relative abundance in liver cirrhosis, NAFLD, and alcohol-associated liver disease (Jiang et al., 2015; Li et al., 2022; Bajaj et al., 2014). However, there have been other studies in late stage chronic hepatitis C (HCV) and HBV patients that show enrichment of the *Faecalibacterium* genus (Aly et al., 2016; Shen et al., 2023).

We additionally identified reduced abundance of several taxa in HDV, including *Flavonifractor*, *Fusicatenibacter, Parasutterella,* and *Parabacteroides*, that have been reported to be diminished in chronic viral hepatitis, advanced liver disease, and alcohol associated liver disease (Jiang et al., 2015; Dubinkina et al., 2017; Aly et al., 2016; Shen et al., 2023; Bajaj et al., 2014). *Flavonifractor*, a member of the SCFA-producing family *Ruminococcaceae*, was depleted in NAFLD and NASH patients (Jiang et al., 2015) and negatively correlated with MELD score (Bajaj et al., 2014). *Parasutterella* was diminished in chronic HBV (Shen et al., 2023), while *Parabacteroides* was decreased in late stage of HCV (Aly et al., 2016).

Several taxa that were enriched in our HDV cohort are also enriched in other liver diseases.

For example, *Collinsella aerofaciens*, which had higher abundance in our study, has also been found in higher abundance in compensated and decompensated cirrhosis (Shao et al., 2018). *Dorea* is enriched in alcohol-dependent individuals with increased intestinal permeability (Leclercq et al., 2014); *Holdemanella* in patients with HCC (Li et al., 2022); and *Lactobacillus* and *Prevotella* in patients with a wide range of liver diseases.

Comparing the gut microbial compositions of the HDV cohort to clinical metadata revealed correlations between the abundance of four genera and HVPG. HVPG is an important surrogate marker for worsening liver disease, with increasing pressures correlating with disease progression. *Prevotella* and *Lactobacillus* were positively correlated with HVPG while *Bacteroides* and *Ruthenibacterium* were negatively correlated with HVPG. *Bacteroides* modulate host energy production through the fermentation of carbohydrates, including host mucosal glycans, into fatty acids, and influence host physiology by altering gene expression, protecting the mucosal barrier, and shaping immune responses (Wexler, 2007). We did not identify correlations between genera and fibrosis score or inflammatory activity. This may reflected by the fact that, in liver disease, changes in portal pressure are detectable before substantial changes in histological fibrosis or inflammatory activity in the liver. Additionally, fibrosis and inflammation scores derived from liver biopsy are subject to sampling variability while HVPG reflects global portal hemodynamics.

In our HDV cohort, we identified a decrease in *Bacteroides* and an increase in *Prevotella* (Nobili et al., 2018; Anderson et al., 2006; Mallick et al., 2021). Several studies have shown that decreased *Bacteroides* are characteristic of obesity (Shao et al., 2018) and alcohol-associated liver disease (Shen et al., 2023). Prior studies have also associated *Prevotella* enrichment with inflammatory states, including in HIV patients, where it has been linked to Th17-mediated T cell activation (Dillon et al., 2016). Th17-mediated immune response plays an important role in the pathogenesis of liver cirrhosis in several viral hepatitides (Paquissi, 2017). Consistent with these observations, increased *Prevotella* abundance has been reported across multiple liver diseases. Experimental studies further support a pathogenic role for *Prevotella*. In mice, *Prevotella* was shown to exacerbate DSS colitis, NASH, and hypertension, and some species have been shown to impair neutrophil function, promoting chronic inflammation (Matsui et al., 2014). Together, these findings suggest that the concurrent increase in *Prevotella* and decrease in *Bacteroides* observed in HDV may contribute to T-cell activation, immune dysregulation, and chronic inflammation.

*Lactobacillus*, from the phylum *Firmicutes*, was also positively correlated with portal pressure, a pattern seen in other liver diseases (Jiang et al., 2015; Nobili et al., 2018; Dubinkina et al., 2017). However, *Lactobacillus* are commonly included in probiotics and have also been studied for their utility in mitigating various diseases, including liver disease, gastrointestinal disorders, metabolic disease, and autoimmune conditions (Jeong et al., 2022). This apparent contradiction suggests the need for more research when considering *Lactobacillus*-containing probiotics in liver disease.

In the metatranscriptome, we found that several cofactor, carrier, and vitamin biosynthesis pathways are upregulated in the HDV cohort. Among the pathways was the gamma-glutamyl cycle which is important for amino acid transport and requires gamma glutamyl transferase (GGT). We found biosynthesis of other pathways such as vitamin B1, B6, B12, B9, and K2, and NAD, FAD, coenzyme A, heme, and S-adenosyl-L-methionine (SAMe) were upregulated, many of which have previously been discussed in the context of liver diseases (Guo et al., 2015; Labadarios et al., 1977). Interestingly, we found upregulation of pyridoxal 5′-phosphate biosynthesis and cycle-related pathways in our HDV cohort. Other studies have reported lower levels of pyridoxal 5′-phosphate in liver disease patients due to increased clearance by the liver. Studies have shown reduction in SAMe synthesis during chronic liver diseases and investigated SAMe supplementation as a possible treatment for various liver diseases (Guo et al., 2015). Our findings of differential expression of pathways related to vitamins, cofactors, coenzymes and metabolism, suggest a possible dysregulation of energy homeostasis in the gut microbiome of the HDV cohort.

We found 29 of the total 194 differentially expressed pathways were related to biosynthesis of amino acids including glutamine, methionine, arginine, histidine, and lysine. Among these pathways, glutamine biosynthesis III showed the highest fold-change of 3.86. Glutamine serves as a key carbon and nitrogen carrier and supports hepatocyte metabolism and proliferation (Paulusma et al., 2022; Lee and Kim, 2019). Gut microbiota-produced glutamine has been shown to alleviate liver injury by promoting metabolic reprogramming of M2 macrophages in the liver (Lu et al., 2023). Arginine, which is necessary for the urea cycle, protein synthesis, and the cell cycle, has been shown to reduce inflammation in alcoholic liver disease, viral hepatitis, and MASLD (Paulusma et al., 2022; Lee and Kim, 2019). Histidine similarly has been shown to reduce inflammatory cytokines in acetaminophen-induced liver injury (Lee and Kim, 2019). Methionine, which is essential for the folate cycle and one-carbon metabolism, has previously been identified as being abnormal in liver cirrhosis (Lee and Kim, 2019). In hepatitis D, where metabolism and inflammatory pathways are disrupted (Khomich et al., 2025), increased amino acid biosynthesis may reflect changes in gut-liver communication that influence host immune responses and metabolic remodeling. Given the roles of amino acids in inflammation, energy metabolism, and cell growth and differentiation, further research is necessary to determine the causes and effects of increased gut microbial amino acid biosynthesis on HDV infection and pathogenesis.

Interestingly, among the 10 most differentially expressed pathways in patients with HDV were superpathways related to degradation of gut mucosal glycans. Degradation of mucosal glycans has been associated with gut barrier dysfunction and increased microbial translocation. Although gut barrier dysfunction is often described as a feature of advanced liver disease, these findings raise the possibility that degradation of gut mucosal glycans may contribute to or precede immune dysregulation in HDV infection.

At the species level, four differentially expressed pathways from *B. fragilis* correlated with Ishak Fibrosis score. These were Isoprene biosysnthesis I, L-lysine biosynthesis III, guanosine ribonucleotides *de novo* biosynthesis, and superpathway of coenzyme A biosynthesis III. Interestingly, several studies have proposed measuring isoprene breath levels as a noninvasive biomarker for liver disease (Mochalski et al., 2023). Another study found that isoprene was higher in portal blood than in the periphery, possibly because it is metabolized in the liver prior to reaching systemic circulation (Mochalski et al., 2023).

There were several limitations to this study. First, the study cohort was of moderate size, comprised of 35 HDV patients and 32 controls. Although sufficient to identify significant microbiome alterations (Ali et al., 2023), the sample size may have limited our ability to detect more subtle associations and may affect the generalizability of findings. Additionally, as the microbiome can be altered by environmental factors, the HCs would ideally come from the same households as the HDV patients to minimize confounding variables. The microbiome can also be influenced by dietary intake. Although we collected dietary information for the day preceding stool collection, dietary intake from the days prior to stool collection was not assessed, which may limit our ability to account for longer-term dietary influences on microbiome composition. Additionally, as the HDV group in this study was composed primarily of individuals in the early stages of disease, we were not able to fully assess the association with disease severity. However, this allowed for the identification of changes associated with earlier stages of disease, giving insight into pathophysiology of disease progression. Also, we were not able to distinguish whether the microbiome changes in patients with HDV could be influenced by HBV. However, all the HDV patients in this cohort were on nucleos(t)ide therapy and had low to undetectable HBV DNA but had positive HDV RNA levels, suggesting that the changes that we see are more likely due to the influence of HDV infection. Future studies are needed to validate these findings and assess the gut microbiome of patients with advanced HDV infection.

In conclusion, this study provided important insights into the gut microbiome in HDV infection, primarily in early disease. As many prior liver-related studies have focused on the gut microbiome in the context of cirrhosis and end-stage disease, it is significant that substantial alterations in the microbiome and its function are present prior to advanced fibrosis. Although this study was observational, these new insights into alterations of microbial energy metabolism in early disease stages provides potential targets for future mechanistic studies investigating how the gut microbiome may contribute to HDV pathogenesis and disease progression. Of note, many of the altered microbes and pathways identified in this study mirror those reported in other liver diseases. Microbial manipulation may therefore be a successful therapeutic option across the spectrum of liver diseases. Given the current understanding of the gut microbiome’s role in liver disease and the lack of effective therapeutics for HDV, these findings will be critical for future studies that aim to improve outcomes in patients with HDV infection.

## Data Availability

The raw data generated in this study can be found in the NCBI (https://www.ncbi.nlm.nih.gov/), accession PRJNA1144402.

## Author disclaimer

The contributions of the NIH author(s) are considered Works of the United States Government. The findings and conclusions presented in this paper are those of the author(s) and do not necessarily reflect the views of the NIH or the U.S. Department of Health and Human Services.

## Supplementary material

SUPPLEMENTARY FIGURE 1 Summary of metagenome assembled genomes (MAGs) detected in the gut of HDV patients and healthy individuals. Total number of MAGs detected, their GC percentage, N50, genomic size, completeness and contamination percentages are depicted in panels **(A–F)**. Completeness of MAGs with respect to its contamination **(G)** and genomic size **(H)**.

SUPPLEMENTARY FIGURE 2 Eight total phyla were identified. Actinobacteria, Bacteroides, and Firmicutes were dominant phyla across all samples.

SUPPLEMENTARY FIGURE 3 There were no significant differences in richness or in Shannon Index between the two groups.

SUPPLEMENTARY FIGURE 4 The UniRef90 gene classification and MetaCyc pathway integration rates of DNA and RNA data (left) and the relative abundance of the top super-classes in MetaCyc pathways.
