# Supplementary figures and images for: Chronic hepatitis D infection is associated with distinguishing microbial and functional features in the gut microbiome
Source: Front Microbiol. 2026 Jul 9;17:1851892. doi: 10.3389/fmicb.2026.1851892 (PMC13393096; doi:10.3389/fmicb.2026.1851892)

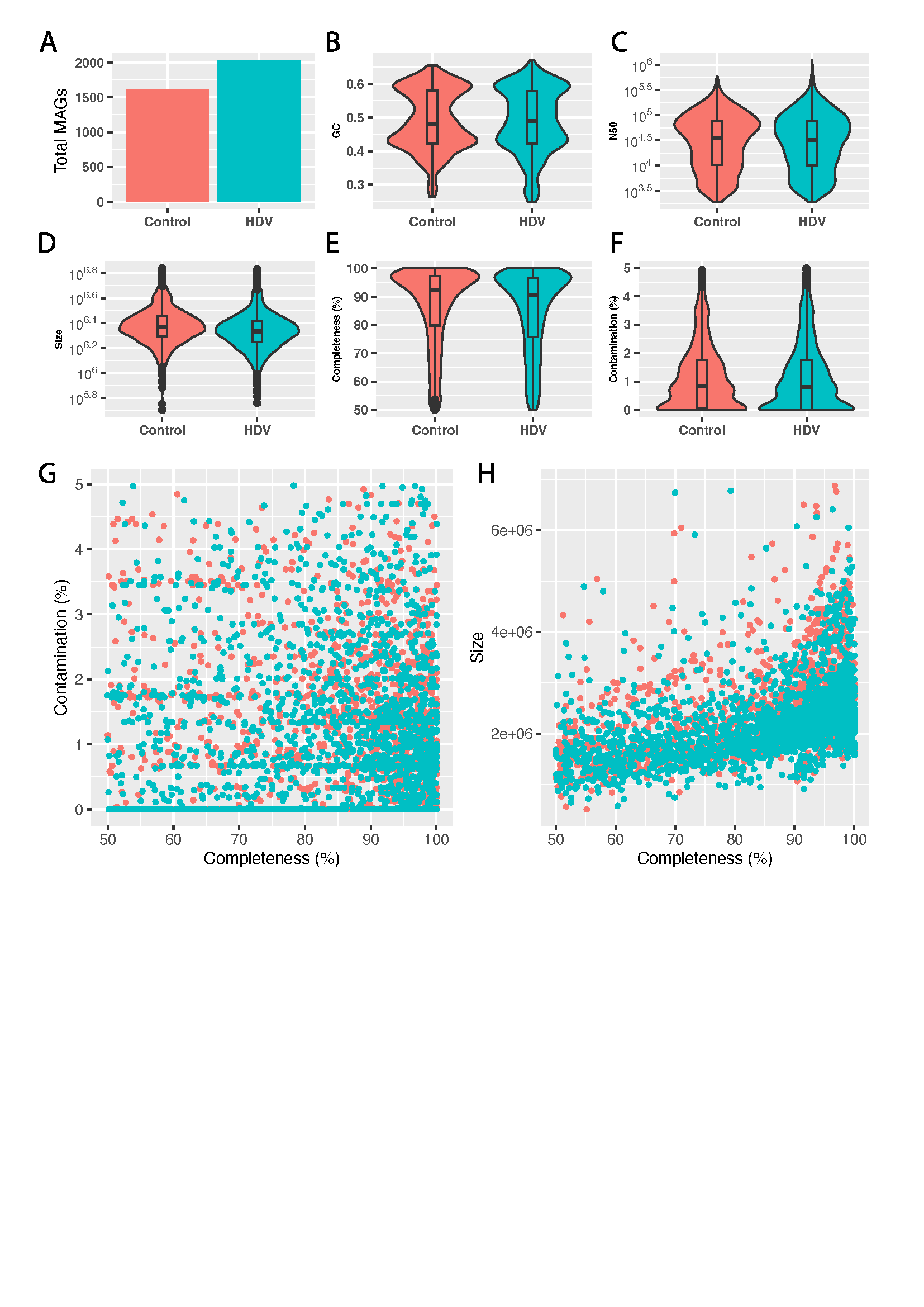

Supplement: SUPPLEMENTARY FIGURE 1 — Summary of metagenome assembled genomes (MAGs) detected in the gut of HDV patients and healthy individuals. Total number of MAGs detected, their GC percentage, N50, genomic size, completeness and contamination percentages are depicted in panels (A–F). Completeness of MAGs with respect to its contamination (G) and genomic size (H). [file Image_1.TIFF]

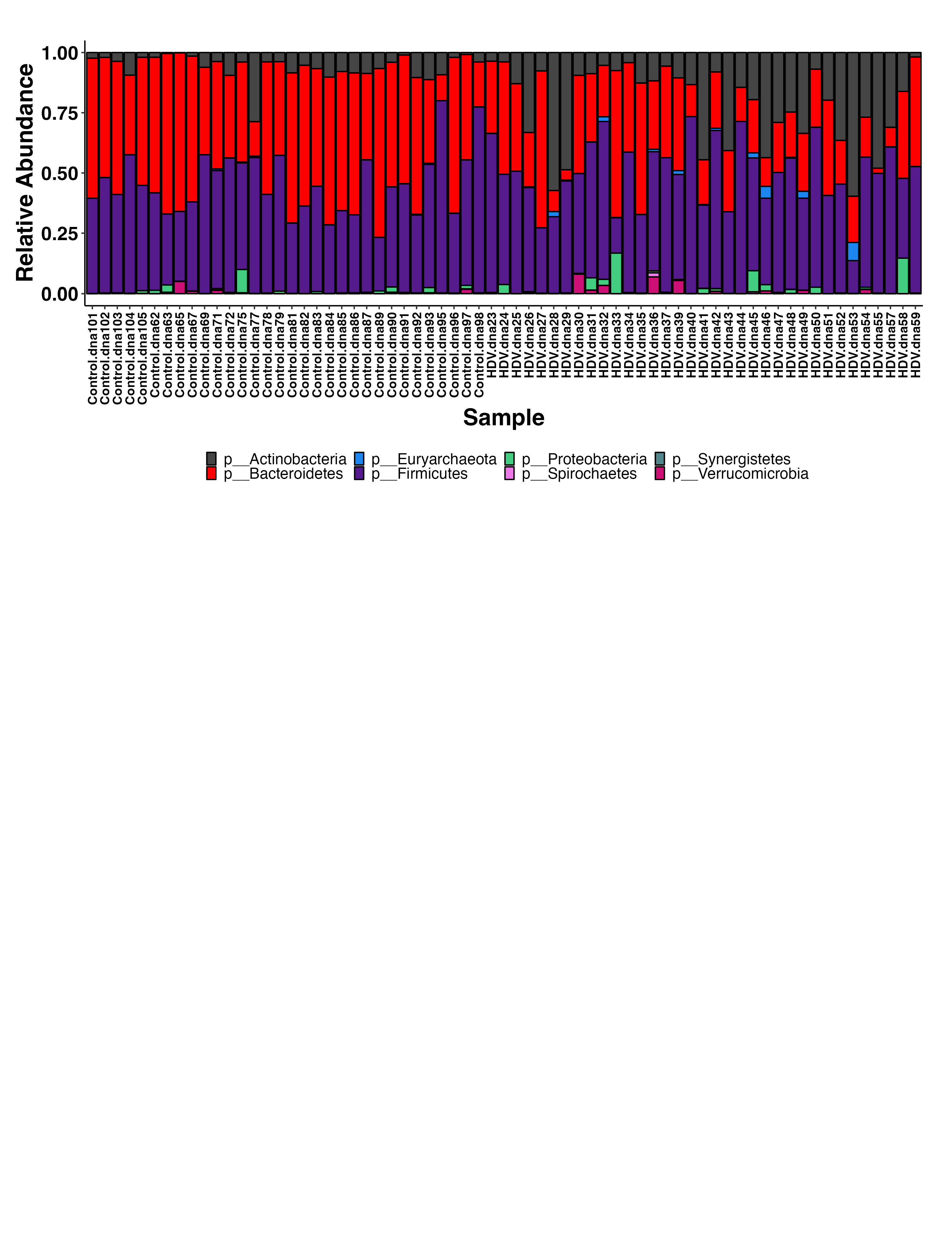

Supplement: SUPPLEMENTARY FIGURE 2 — Eight total phyla were identified. Actinobacteria, Bacteroides, and Firmicutes were dominant phyla across all samples. [file Image_2.TIFF]

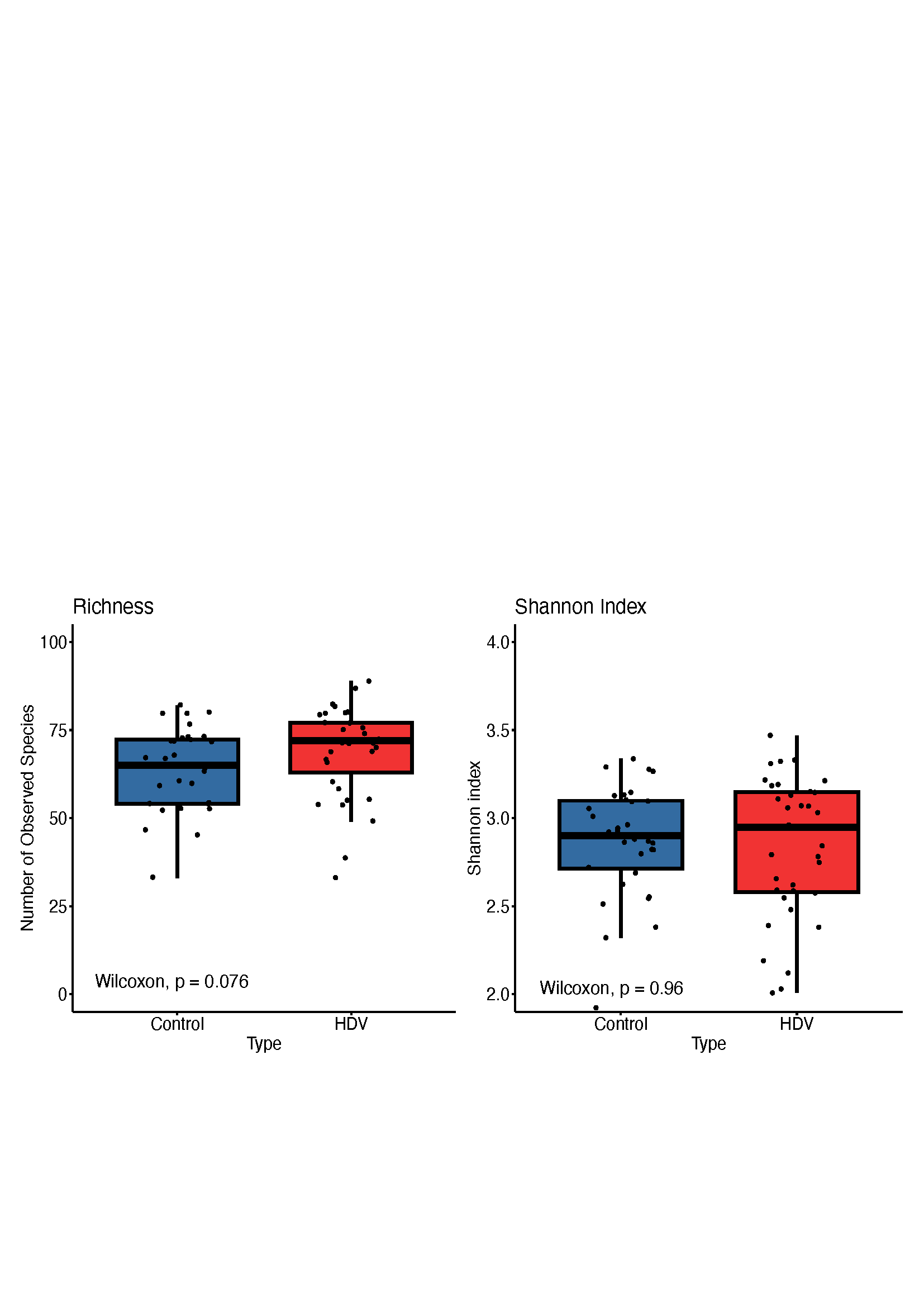

Supplement: SUPPLEMENTARY FIGURE 3 — There were no significant differences in richness or in Shannon Index between the two groups. [file Image_3.TIFF]

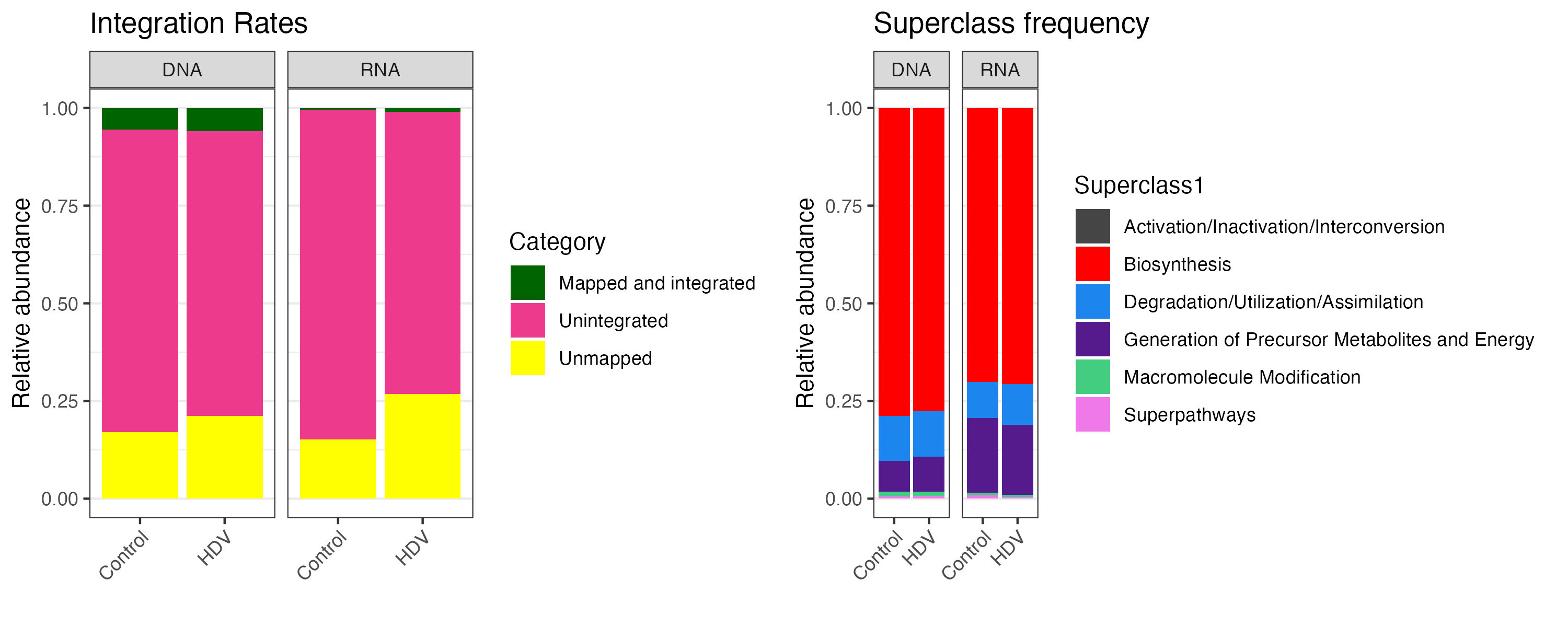

Supplement: SUPPLEMENTARY FIGURE 4 — The UniRef90 gene classification and MetaCyc pathway integration rates of DNA and RNA data (left) and the relative abundance of the top super-classes in MetaCyc pathways. [file Image_4.JPEG]
